# The Antinutritional Factors and Technological Processing of Sorghum and Its Application in Pig Production

**DOI:** 10.3390/ani15030328

**Published:** 2025-01-24

**Authors:** Jianjian Zhang, Ping Li, Xuefen Yang, Li Wang

**Affiliations:** State Key Laboratory of Swine and Poultry Breeding Industry, Key Laboratory of Animal Nutrition and Feed Science in South China, Ministry of Agriculture and Rural Affairs, Lingnan Modern Agricultural Science and Technology Guangdong Laboratory Maoming Branch, Guangdong Key Laboratory of Animal Breeding and Nutrition, Institute of Animal Science, Guangdong Academy of Agricultural Sciences, Guangzhou 510640, China; zjgsau@163.com (J.Z.); yangxuefen@gdaas.cn (X.Y.)

**Keywords:** sorghum, antinutritional factors, nutrient value, pig

## Abstract

Sorghum ranks as the fifth largest grain crop globally, and it has similar levels of crude protein and leucine compared to corn, making it a viable substitute for full or partial corn in pig diets. Sorghum is produced around the world like corn, which is the energy source in pig diets. However, sorghum contains antinutritional factors that adversely affect nutrient absorption, energy, and amino acid availability. Additionally, the low content of essential amino acids, such as lysine (Lys) and methionine (Met), limits its feeding value. Consequently, this paper primarily discusses the antinutritional factors present in sorghum and elaborates on methods to enhance the nutritional value of forage sorghum through processes such as crushing, extrusion, and enzymatic hydrolysis. The nutritional value of sorghum could be improved by adding protease, by extrusion, and by reducing the grain size. Furthermore, this paper summarizes the application of sorghum in pig production to enhance the understanding of forage sorghum among feed enterprises and to provide a theoretical reference for the rational formulation of pig diets.

## Simple Summary

This review explores the potential of sorghum as an alternative to corn in swine diets, focusing on summarizing the current state of knowledge regarding the role of sorghum in animal nutrition and expounding the improvement of nutritional value of forage sorghum by crushing, extrusion, and enzymatic hydrolysis. It highlights the antinutritional components that are present and the processing methods affecting the reduction in their content, such as a low essential amino acid content and high levels of antinutritional compounds. It organizes information on the nutritional value of sorghum as feed and the possibilities of its use in the nutrition of pigs. It has a certain reference function for professionals engaged in pig nutrition, feed raw materials, and feed resources development.

## 1. Introduction

**Figure 1:**
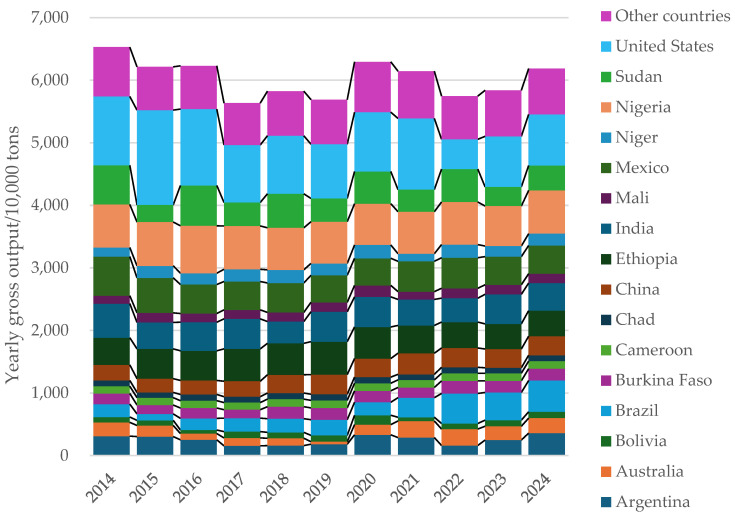
Sorghum production by country in the most recent 10 years.

In animal production, it has become important to necessitate the exploration and development of alternative ingredients with cost-effective potential, due to feed ingredient prices fluctuating, particularly when the prices of traditional feed ingredients such as corn rise [1]. Consequently, it is crucial to explore and develop effective, low-cost feed ingredients to substitute for corn and wheat as energy sources in swine diets. Sorghum presents a feed ingredient with potential and nutritional value, which can be used primarily as a source of feed for swine [2]. According to official data from the National Bureau of Statistics (NBS), the total Chinese production of sorghum in 2023 was 3.64 million tons, with animal feed estimated to be approximately 1.8 million tons, representing about 49.5% of the total production [3]. The output of sorghum by country in the past 10 years is shown in Figure 1. Compared with corn and wheat, sorghum contains similar levels of crude protein (CP), energy, and amino acids (such as proline (Pro), leucine (Leu), alanine (Ala), and glutamic (Glu)) [4]. However, antinutritional factors in sorghum, such as tannins and kafirin, usually reduce the utilization of nutrients, energy, and amino acids, with a low content of limiting amino acids such as Lys and Met being present, thereby constraining the feeding value of sorghum [5]. Nonetheless, treatments such as smashing, extrusion expansion, and enzymatic digestion can enhance the nutrient, energy, and amino acid utilization of sorghum by pigs [6]. Therefore, the various species of sorghum antinutritional factors, the different treatment processes, and their applications in swine feed formulations will be reviewed in this study, aiming to provide theoretical references for the rational formulation of pig feed rations and to maximize the potential applications of sorghum in swine diets.

## 2. Antinutritional Factors of Forage Sorghum

### 2.1. Distribution and Structure

**Figure 2:**
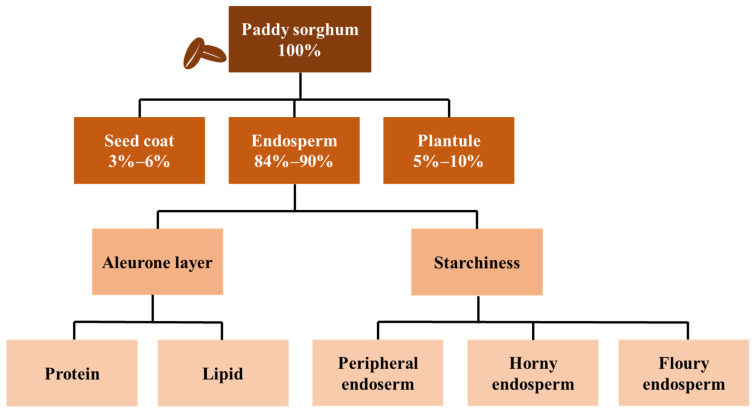
Composition of sorghum seed.

The characteristics of sorghum grains, including the shape, size, color, and hardness, are closely related to the specific varieties of sorghum. Sorghum grains consist primarily of the seed coat, endosperm, and germ (Figure 2). The hardness and texture of the grain are related to the endosperm [7]. There are variations of sorghum depending on the region and cultivar: sorghum is available in white, red, brown, and black [8]. As an energy feed ingredient, the nutritional composition of sorghum is similar to that of corn and wheat [9]. In recent years, advancements in sorghum varieties have led to an increase in energy content from 96% to 98–99% (relative to the energy content of corn), while the levels of the antinutritional factors have gradually decreased [10,11]. Compared with corn and wheat, sorghum has similar concentrations of gross energy (GE), CP, and ether extract (Table 1) [12]. Consequently, the feeding value of sorghum is becoming closely comparable to that of corn and wheat. However, the nutritional composition of sorghum varies significantly due to differences in the planting conditions and varieties, resulting in diverse applications in diets across different countries. For instance, approximately 25% of the sorghum produced in the United States is utilized for feed and typically exhibits a low tannin content. In contrast, Chinese sorghum, which has a higher tannin content, is predominantly used for brewing. The tannin content in feed sorghum is generally around 5%.

### 2.2. Tannins

**Figure 3:**
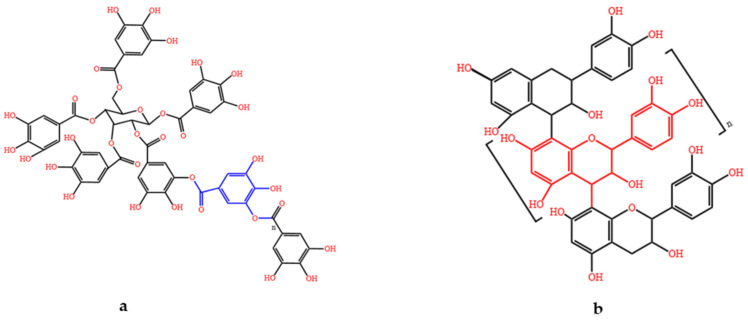
The composition of tannins [14]: (**a**) hydrolysable tannin; and (**b**) condensed tannin.

Tannins, also known as ellagic acid or tanning agents, are important secondary metabolites in plants [13]. Tannins in sorghum can be classified as condensed tannins and hydrolysable tannins on the basis of their chemical composition (Figure 3) [14]. Condensed tannins, known as proanthocyanidins, are polymers of flavan-3-ols, with their basic units linked by C-C or C-O-C bonding [15]. Flavan-3-ols possess a typical C_6_-C_3_-C_6_ flavonoid skeleton, denoted by the letters A, B, and C [15]. The differences among these structures depend mainly on the number of hydroxyl groups on the aromatic ring and the spatial structure of the asymmetric carbons on the heterocyclic ring [15]. Hydrolyzed tannins, also known as gallic acid, are phenolic compounds formed by the polymerization of polyols and sugars with phenolic acids and their derivatives through ester bonding; on the basis of their structure, they can be divided into gallic tannins and ellagitannins, both of which exhibit some antioxidant effects in animals [16]. The primary factor affecting the digestion and absorption of sorghum in pigs is condensed tannins, which can polymerize under acidic or alkaline conditions to form substances that are not easy to digest, thereby exerting an antinutritional effect [17]. Typically, the darker the color of the sorghum grain, the greater its tannin content; however, this method of assessment is not very reliable, because the color of sorghum grain is influenced by various factors, such as the color of the exocarp, the thickness of the mesocarp, and the presence or absence of the seed coat [18].

The content of the tannins is a significant factor affecting the available energy and digestibility of the protein in sorghum [19]. Compared with corn, sorghum is a source of dietary energy for pigs without any adverse effects on the digestive enzyme activities or growth performance [4,20]. In contrast, high-tannin sorghum has lower activities of antioxidant and digestive enzymes, which reduces nutrient utilization [21]. It is reported that the tannin content in the sorghum fed to growing pigs negatively affects the standardized ileal digestibility (SID) values of amino acids, including Lys, threonine (Thr), valine, arginine (Arg), serine (Ser), and aspartic (Asp) [22,23]. Endogenous proteins are rich in Thr, Arg, Ser, Glu, and Asp [24,25]. Tannins can form complexes with proteins and endogenous enzymes in the feed, hindering the hydrolysis of proteins. This results in the reduced digestibility of the starch and protein, as well as an increased loss of endogenous proteins (enzymes and mucins) in the small intestine [17,26]. Furthermore, tannins stimulate the intestine, resulting in increased mucus secretion and protein binding, which decreases the digestibility of Thr and Arg; the precipitation of globular proteins (albumin and globulin) leads to a reduction in Lys digestibility [27]. Consequently, when high-tannin sorghum is utilized as swine feed, supplementation with extra protein or amino acids may be necessary to satisfy the amino acid requirements [28]. However, negative effects on the SID of amino acids are likely to occur only when the tannin content exceeds 1% [22].

### 2.3. Kafirins

Kafirins are the major components of the sorghum endosperm, with diameters ranging from 0.4 to 2.0 μm [29]. These proteins constitute up to 68% to 73% of the total protein in sorghum and 77% to 82% of the total protein in the endosperm [30]. The distribution of amino acids in the sorghum kafirin is unbalanced (Table 2) [31], with Glu, Ala, Leu, and Pro comprising more than 65% of the total amino acid content and Lys, Met, Thr, and tryptophan present at relatively low levels [32]. Consequently, the presence of kafirin significantly limits the feeding value of sorghum for swine. These proteins are categorized as α-, β-, γ-, and δ-kafirin, according to their amino acid composition, sequence, molecular mass, and other factors [31,32,33,34]. The amino acids contained in kafirin varied greatly among the different sorghum varieties (Table 3). On the one hand, there were different varieties of sorghum and different detection methods for kafirins; on the other hand, the number of test samples was relatively small and there could have been experimental errors caused by human manipulation in the testing process; therefore, it is necessary to carry out the tests more carefully in order to obtain more accurate experimental data, which will provide data support for the study of kafirins in sorghum. The α-kafirin is the main structure, accounting for approximately 70% to 80% of the total, and is easily digested; however, it is encapsulated by β-kafirin and γ-kafirin, which are resistant to digestion and form disulfide bonds that reduce protein digestibility [7]. Specifically, β-kafirin accounts for 7% and γ-kafirin for 12%, and δ-kafirin has the lowest content at 1% [35]. The endosperm structure of sorghum is composed of horny endosperm and powdered endosperm in a certain proportion [36]. The starch granules in the powdered endosperm are more loosely bound to the proteins and are easily digestible, whereas the starch granules in the horny endosperm are tightly wrapped by kafirin and embedded within the gluten matrix, making them more difficult to digest [37]. Chen et al. [38] assessed the digestibility of 31 sorghum samples, and the results indicated that a lower kafirin content in sorghum corresponds to a greater availability of key amino acids, such as Lys and Met. This finding shows that, within the protein composition of sorghum, the amino acid availability value of kafirin is lower than that of non-kafirin.

### 2.4. Phytates

**Figure 4:**
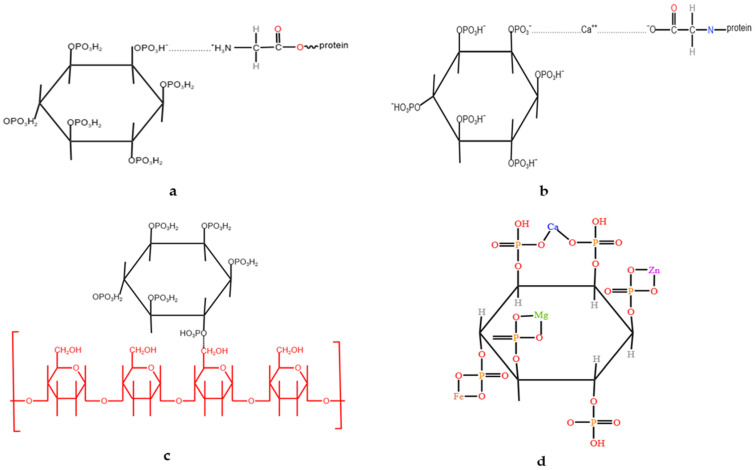
Phytate in pig and poultry nutrition [41,43,44]. (**a**) Phytic acid–protein complex at low pH. (**b**) Phytic acid–protein complex at neutral pH. (**c**) Interaction of phytic acid with starch. (**d**) Phytic acid–chelat at neutral pH.

Phytate is an organic acid in sorghum grains that exists mainly in the form of 6-inositol phosphate and a cyclic compound containing six phosphate groups [39]. Phytic acid, which can exist as a free acid, is unstable and has the capacity to form phytate chelates with various metal cations, such as calcium, iron, zinc, magnesium, potassium, and manganese (Figure 4d) [40,41]. These phytate complexes bind to protein starch, reducing protein solubility and enzyme activity (Figure 4a–c) [42,43,44]. Cowieson et al. [45] reported that phytate significantly increases endogenous Na^+^ secretion in the small intestine. The entry of Na^+^ into the small intestine, via the Na^+^ transport system and Na^+^/K^+^-ATP, may influence the intestinal absorption of the feed and endogenous amino acids, while also inhibiting amino acid absorptions [46].

Additionally, phytic acid inhibits the activity of various digestive enzymes, including amylopectin hydrolases, lipases, and protein hydrolases, thereby impacting the digestion and absorption of starch, fat, and proteins [47,48]. Doherty et al. [49] demonstrated that phytate phosphorus in sorghum grains is primarily concentrated in the seed coat, with a content ranging from 0.57% to 1.69%. Selle et al. [50] evaluated 15 different varieties of sorghum and reported total phosphorus and phytate phosphorus contents ranging from 0.205% to 0.430% and 0.17% to 0.37%, respectively, where nearly 66% to 93% of the phosphorus was found in the form of phytate phosphorus. Krasucki et al. [51] added phytase to sorghum feed and increased the digestibility of CP, amino acids, and organic matter in sows by 25%, 2%, and 7%, respectively. These results indicate that the inclusion of phytase is beneficial for increasing the digestibility of amino acids and starch in sows.

## 3. Treatment Processes to Improve Sorghum Feeding Value

### 3.1. Enzymes

Enzyme preparations are specific and highly efficient biocatalysts derived from animals, plants, or microorganisms, and they are widely utilized in livestock and poultry production worldwide [52]. These enzyme preparations not only compensate for deficiencies in digestive enzymes within the animal body but also increase the activity of enzymes, thereby improving the overall health status of the organism [53]. Furthermore, enzyme preparation plays a role in reducing the antinutritional factors present in feed and enhancing feed utilization efficiency [54].

Arabinoxylan and cellulose are the more abundant fiber components found in the dietary fiber fraction of grains and grain by-products [55]. The contents of arabinoxylan and cellulose in corn and sorghum were 480 g/kg and 440 g/kg and 210 g/kg and 220 g/kg, respectively [56]. Additionally, sorghum has a relatively low concentration of soluble non-starch polysaccharides (NSPs), resulting in reduced microbial fermentation at the distal end of the large intestine [57]. The addition of xylanase enhances the concentration of arabino-oligosaccharides in the digestive tract of weaned piglets fed sorghum basal diets [58]. These oligosaccharides can be used as substrates for gut microbial fermentation, thereby stimulating fermentation and potentially improving nutrient digestibility and feed utilization [59,60]. Oliveira et al. [61] incorporated xylanase and β-glucanase enzyme blends into sorghum rations and reported that the addition of these enzyme blends increased the GE apparent total tract digestibility (ATTD), digestible energy (DE), and metabolizable energy (ME) in growing pigs by approximately 2.5% (*p* < 0.05), 2.7% (*p* < 0.05), and 3.0% (*p* > 0.05), respectively. In the sorghum endosperm, starch granules are encapsulated by kafirin and cell walls, which influence the digestibility of the amino acids and starch, as well as the efficacy of the enzymes [62]. González-Ortiz et al. [58] investigated the impact of xylanase supplementation in feed rations on the growth performance and gut flora of piglets and reported that the addition of xylanase to the diets of sorghum-fed pigs improved nutrient digestibility and promoted arabinoxylan release. Oliveira et al. [63] added xylanase and cellulase to sorghum–soybean meal DDGS and sorghum–soybean meal wheat diet rations, which increased the apparent digestibility of GE in growing pigs by 2.6% and 3.2% (*p* > 0.05), DE and ME by 2.7% and 3.2% (*p* > 0.05), and ME by 3.0% and 1.5% (*p* > 0.05), respectively.

Sorghum has poor protein digestibility due to the hydrophobicity and disulfide crosslinking bonds of kafirins [64]. Keratinases, a class of protein hydrolases, can cleave disulfide bonds and hydrolyze soluble casein, insoluble keratin, and other proteins crosslinked by disulfide bonds [65]. The supplementation of keratinase into sorghum basal diets can hydrolyze kafirin, thereby increasing protein digestibility [66]. Chen et al. [66] investigated the effects of adding protease to sorghum basal diets on the growth performance and intestinal health in pigs. Their findings indicated that the inclusion of protease increased CP digestibility in the duodenum by 5% (*p* < 0.05), reduced the malondialdehyde (MDA) concentration in the duodenum by 18.3% (*p* < 0.05), and improved the ratio of the villus height to the crypt depth from 1.06 to 1.21 (*p* < 0.05), likely by reducing oxidative stress to promote pig growth and feed intake. Peng et al. [67] examined the effects of protease on growth performance and nutrient digestibility in the gut of sorghum diet growing pigs, and their results showed that the addition of 400 mg/kg of protease to the diet increased the growth performance of growing pigs, potentially due to improvements in the nutrient digestibility, host metabolism, immune status, and gut flora distribution.

Trindade et al. [68] added carbohydrase and phytase to diets to study the effects of enzymes on sorghum. They found that the addition of carbohydrase increased the sorghum dry matter (DM) by 3.3% (*p* < 0.05) and the apparent whole gut digestibility of ME by 1.4% (*p* > 0.05); the mix of carbohydrase and phytase resulted in increases of 5.8% in the DM (*p* > 0.05), 6.4% in CP (*p* > 0.05), and 4.6% and 4.1% in the apparent whole gut digestibility of digestible energy and ME (*p* > 0.05), respectively. The degradation of NSPs by enzymes not only increases cell wall solubility but also has the potential to enhance the effectiveness of phytase and improve phosphorus release from phytates [69].

### 3.2. Physical Processing Treatment

Heat treatment can alter the structure of proteins, extend their molecular arrangement, and increase the contact area between digestive enzymes and proteins [70]. An appropriate temperature can augment the degree of gelatinization and the hardness of starch, improving feed digestibility [71]. However, high temperatures may trigger the Maillard reaction in amino acids, which can increase the hardness of the pellet feed and adversely affect animal digestion [72]. Moreover, high temperatures may disrupt the noncovalent interactions among proteins, leading to structural changes and the formation of disulfide bonds between β-kafirin and γ-kafirin, which encase α-kafirin and expose hydrophobic amino acid residues within the proteins, decreasing protein solubility [73]. Conversely, low temperature can reduce the degree of starch gelatinization, resulting in insufficient feed viscosity, poor pellet quality, decreased protein digestibility, and increased incidence of diarrhea in pigs [74]. Notably, sorghum generally has a greater degree of gelatinization than corn and wheat [75]. Rooney et al. [76] indicated that starch digestibility in sorghum diets decreases at 85 °C, which may increase the resistant starch content at this temperature, and that high temperatures may reduce the interactions between starch and proteins, leading to the disruption of the chemical bonds within the starch granules. Wang et al. [77] examined the impact of various temperatures (65, 70, 75, 80, and 85 °C) on nutrient digestibility and the intestinal flora of sorghum diets and reported that the average daily gain (ADG) was highest at 75 °C and 80 °C, with values of 988 g and 985 g, respectively; the feed conversion ratio (FCR) was 2.317 and 2.328, respectively; and the diarrhea rates were 1.695% and 1.797%, respectively. Increasing the temperature from 75 °C to 80 °C resulted in the number of beneficial bacteria, specifically *Bifidobacterium* and *Lactobacillus*, and the apparent digestibility of CP and starch in the ileum reaching their peak at 75 °C and 80 °C, with values of 87.84% and 87.95% for CP and 92.26% and 91.58% for starch, respectively.

Cabrera et al. [78] investigated the effects of different particle sizes (800, 600, and 400 µm) of sorghum on the performance and apparent digestibility of growing pigs and reported that the highest nutrient digestibility occurred at a size of 400 µm (*p* < 0.05). Healy et al. [79] reported that reducing the sorghum particle size from 900 µm to 300 µm resulted in an increased ADG in pigs (*p* < 0.05). Owsley et al. [80] examined the impact of grain size on the performance and apparent digestibility of growing pigs and reported that, when the sorghum grain size was decreased from 1262 µm to 471 µm, the apparent ileal digestibility (AID) of the pigs improved, with increases of 12.2% (*p* < 0.05) for the DM, 16.0% for starch (*p* < 0.05), and 12.6% (*p* < 0.05) for GE. Ohh et al. [81] also found that smashing the sorghum seed grain increased nutrient digestibility. Additionally, Paulk et al. [2] researched the effects of different sizes (target sizes: 800, 600, and 400 µm; actual sizes: 724, 573, and 319 µm) on the growth performance of growing pigs. They found that, although reducing the sorghum particle size from 724 µm to 319 µm did not affect the ADG (*p* > 0.05), the FCR increased by 3.7% (*p* < 0.05).

Grain extrusion has been shown to enhance starch digestibility and growth performance in weaned piglets [82]. Extrusion with heat and water can enhance the contact area of enzymes with the grain and induce the gelatinization of the starch granule [83]. The proanthocyanidins (condensed tannins) present in sorghum are bound to fibers, proteins, and minerals and the depolymerization of proanthocyanidins during extrusion leads to the degradation of proanthocyanidins and fibers during processing, thereby increasing nutrient digestibility [84]. Rodriguez et al. [85] investigated the impact of extrusion on the energy and nutrient digestibility of swine diets and reported that extruded sorghum increased the ATTD of GE by 6.7% (*p* < 0.05), DE by 6.0% (*p* < 0.05), ME by 4.9% (*p* < 0.05), the AID of starch by 5.8%, and the SID of CP by 5.5% (*p* < 0.05). Although extruded sorghum improved the digestibility of nutrients such as GE and CP, it did not significantly affect the average daily weight gain or the FCR [86]. Zhang et al. [87] determined the effective energy digestibility and nutrient digestibility of expanded sorghum, revealing that the SID ranged from 9.5% to 28.3% for all the amino acids, with the exception of Leu, and found an interaction between seed type and extrusion treatment for the SID of amino acids in growing pigs (*p* < 0.05). Furthermore, extrusion puffing sorghum prior to pelleting may enhance nutrient digestibility in pigs [2]. While sorghum enhances digestibility and decreases antinutritional factors when extruded and milled, the increased costs and economic losses associated with processing were not addressed in the various studies. Moreover, no tannase was found when dietary enzyme preparations were added, so these problems are still worthy of further study. The effects of sorghum processing technology on pig production performance are shown in Table 4.

## 4. Application of Sorghum to Swine Diets

### 4.1. Effects of Feeding Sorghum on Swine Performance and Nutrient Digestibility

Fully or partially replacing corn with sorghum can be used without compromising the growth performance of pigs [86]. The growth performance of pigs fed different amounts of sorghum is shown in Table 5 [4,20,66,86,88,97,98]. However, it has been reported that replacing corn with sorghum in the diet of lactating sows results in a slight reduction in feed intake and litter weight gain [10]. Song et al. [99] investigated the substitution of corn with 30%, 65%, and 100% sorghum, and found that replacing 65% and 100% corn with sorghum increased antioxidant enzyme activities by 32.4% and 38.7% (*p* < 0.05), respectively, while lowering the MDA concentrations in pigs (*p* < 0.05). The findings indicate that the substitution of corn with sorghum enhances the serum antioxidant capacity of pigs. Cousins et al. [100] reported that, after replacing corn with high-tannin sorghum, the FCR, ileal digestibility of nitrogen, and GE decreased in growing pigs (50 kg BW) and that the use of low-tannin sorghum in diets did not impact growth performance. Pan et al. [97] divided 144 weaned piglets into four groups, with the ratios of sorghum to corn in their diets designed at 0:60, 20:40, 40:20, and 60:0; this research indicated no significant differences in the ADG and average daily feed intake (ADFI) between the groups. The inclusion of 60% sorghum resulted in a decrease in the apparent whole gut digestibility of CP from days 15 to 28 (*p* < 0.05). Furthermore, the average daily weight gain of finishing pigs increased without a significant change in the FCR when they were fed sorghum basal diets [2]. Various factors may affect growth performance such as the growth stage of the pigs, the varieties of sorghum, the growing environment of the sorghum, and differences in the tannin and kafirin contents. Furthermore, while many experiments have investigated the appropriate proportion of sorghum to replace corn in swine feed rations, the rationale for the selection of the experimental sorghums is often not provided. This omission can result in data that may be misleading for practical production applications, particularly when samples with a high nutrient content are deliberately chosen. On the other hand, when evaluating the effects of sorghum with varying tannin contents in swine rations, the selection of different sorghum varieties and planting environments for experimental studies can inevitably introduce errors into the test results.

### 4.2. Effects of Feeding Sorghum on the Intestinal Health of Pigs

Chen et al. [101] reported that the inclusion of 65–72% sorghum in feed resulted in a reduction in the MDA levels in the serum and jejunum of swine by 55.6% and 35.9% (*p* < 0.05), respectively, and the jejunal villus height and crypt depth decreased by 19.5% and 17.1% (*p* < 0.05), respectively. The replacement of corn with sorghum led to increases in the average daily weight gain and ADFI of 4.0% and 6.7% (*p* < 0.05), respectively, whereas the FCR decreased by 2.99% (*p* < 0.05). The findings suggest that the use of sorghum to fully replace corn in diets could benefit pigs with increased growth and feed intake, potentially by reducing oxidative stress. Furthermore, Chen et al. [66] demonstrated that replacing corn (sorghum accounted for 65% of the total) with sorghum resulted in an increase in the ADG and daily feed intake of 4.7% and 1.3% (*p* < 0.05), respectively, whereas the FCR decreased by 3.6% (*p* < 0.05). The addition of sorghum to the rations lowered the MDA content in the pig serum and jejunum by 56.9% and 28.6% (*p* < 0.05), respectively, along with reductions in the jejunal villus height and crypt depth of 21.7% and 23.0% (*p* < 0.05), respectively. The inclusion of sorghum in the diets of weaned pigs may alleviate diarrhea and oxidative stress, significantly lowering the MDA content in the serum and jejunum [97,101]. Additionally, it reduces the villus height and crypt depth in the jejunum, while increasing the population of beneficial bacteria, such as *bifidobacterium* and *lactobacillus*, in the intestine [101,102]. This dietary inclusion also improves the feed intake and promotes the growth of pigs. It is recommended that pigs be fed diets containing less than 20% sorghum during the first two weeks and no more than 40% during the subsequent two weeks post-weaning [97].

### 4.3. Effect of Feeding Sorghum on Pork Quality

Pigs fed a diet with sorghum had a more balanced proportion of fatty acids in their back fat and improved meat quality than pigs fed corn basal diets [86]. Zhang et al. [103] found that replacing corn with sorghum at levels of 30%, 65%, and 100% resulted in increases in back fat thickness of 3.0%, 5.2%, and 4.3% (*p* < 0.05), respectively. The contents of oleic acid, trans-oleic acid, arachidic acid, and arachidonic acid in the longissimus dorsi increased by 15.3%, 39.6%, 56.8%, and 52.4%, respectively, following 100% corn replacement (*p* < 0.05). The contents of oleic acid, trans-oleic acid, arachidic acid, and arachidonic acid in the longissimus dorsi increased by 37.9%, 77.15%, 72.2%, and 53.2%, respectively, in the 65% replacement group (*p* < 0.05). These findings suggest that sorghum improves meat quality when it is partially replaced with corn in the diet.

## 5. Conclusions

Sorghum is used as one of the energy feedstocks to replace corn in pig diets, because it contains nutrients similar to corn, such as crude protein, energy, some amino acids, etc. Nevertheless, the use of sorghum as pig feed has some limitation due to the presence of antinutritional factors. Thus, treating sorghum by some treatments, such as grinding, heat treatment, extrusion, expansion, and enzyme preparation, removes and/or reduces these antinutritional factors, thereby improving sorghum quality. Extrusion and grinding can improve the sorghum quality and product quality, but enzyme preparation, expansion, and other treatment methods need to be further studied. As a result, it is recommended to further study the biological mechanism and specific enzyme preparations, in order to explore the promoting effect of sorghum in pigs and the best method of processing.

## Figures and Tables

**Table 1:** Proximate composition of sorghum, corn, and wheat (as–fed basis) [12].

Items	Sorghum	Corn	Wheat
Dry matter/%	89.39	89.31	86.38–88.67
Gross energy/(kcal/kg)	3881	3933	3788–4295
Digestible energy/(kcal/kg)	3596	3451	3313–3450
Metabolizable energy/(kcal/kg)	3532	3395	3215–3376
Net energy/(kcal/kg)	2780	2672	2472–2595
Crude protein/%	9.36	8.24	10.92–14.46
Ether extract/%	3.42	3.48	1.36–1.82
Crude fiber/%	2.14	1.98	2.57
Neutral detergent fiber/%	10.63	9.11	10.60
Acid detergent fiber/%	4.93	2.88	3.55
Ash/%	1.64	1.30	1.98–1.99
Ca/%	0.02	0.02	0.03–0.06
P/%	0.27	0.26	0.30–0.39
Essential amino acids (%)			
Arginine	0.36	0.37	0.52–0.60
Histidine	0.21	0.24	0.28–0.34
Isoleucine	0.36	0.28	0.34–0.47
Leucine	1.21	0.96	0.68–0.91
Lysine	0.20	0.25	0.35–0.39
Methionine	0.16	0.18	0.22
Phenylalanine	0.48	0.39	0.52–0.64
Threonine	0.30	0.28	0.35–0.40
Tryptophan	0.07	0.06	0.14–0.17
Valine	0.46	0.38	0.47–0.58

**Table 2:** Comparison of the properties of the four types of kafirin protein [31].

Kafirin Type	Molecular Mass	No. of Amino Acid Residues	Amino Acid Composition	Polymerization Behavior	Number of Genes
α	26,000–27,000	240–250	Rich in non-polar amino acids; no Lys; one Trp; 10 blocks of repeated amino acids	Monomers, oligomers, and polymers	About 20
β	18,745	172	Rich in Met and Cys, two Trp	Monomers and polymers	1
γ	20,278	193	Rich in Pro, Cys, and His; no Lys, Asn, Asp, and Trp	Oligomers and polymers	1/2
δ	12,961	114	Rich in Met; no Lys; one Trp	No	No

**Table 3:** Amino acid composition of kafirin/% (as–fed basis) [32,34].

Amino Acid	1	2	3	4	5	6	7	8	9
Lys	0.1	0.1	0.3	0.34	0.21	0.25	0.30	0.27	0.22
Met	2.1	1.0	1.1	0.94	0.91	1.00	1.03	1.28	0.96
Thr	2.9	2.6	2.8	2.02	2.06	1.93	1.91	1.94	2.03
Val	3.8	5.0	5.1	3.26	3.46	3.01	3.19	3.56	3.39
Ile	3.0	4.8	4.2	3.07	3.20	2.87	2.96	3.28	3.28
Leu	17.5	19.2	18.8	12.17	13.05	11.53	11.57	12.84	13.15
Arg	3.8	1.0	2.0	1.33	1.18	1.23	1.24	1.16	1.18
His	1.6	0.9	1.3	1.35	1.40	1.32	1.33	1.05	1.23
Phe	6.6	6.4	4.6	4.27	4.42	4.05	4.04	4.54	4.63
Ala	11.8	12.4	12.5	7.89	8.56	7.49	7.64	8.57	8.49
Asp	6.0	6.5	7.3	4.17	4.38	3.80	3.83	4.43	4.52
Cys	3.2	0.4	0.7	0.29	0.37	0.41	0.54	0.63	0.36
Glu	28.2	30.0	30.5	18.86	20.37	17.86	17.77	19.53	20.27
Gly	1.4	1.1	1.2	1.24	1.10	1.19	1.21	1.04	1.10
Pro	10.2	10.0	10.1	9.21	9.74	9.25	9.03	8.81	9.58
Ser	4.3	4.1	3.9	3.28	3.37	3.13	3.14	3.35	3.36
Tyr	3.6	5.5	5.3	3.57	3.75	3.41	3.33	3.67	3.80

**Table 4:** Effect of sorghum processing technology on pig performance.

Items	Technology Processing	Body Weight (kg)	Result	References
Enzymic preparations	multicarbohydrase preparation (700 U of α-galactosidase, 2200 U of galactomannanase, 3000 U of xylanase, and 22,000 U of β-glucanase)	6.2 ± 0.35	Polysaccharide enzyme preparation can increase DM digestibility and the ME/GE of sorghum.	[68]
	phytase (500 FTU)	18.6, 22.3 ± 1.8	After adding phytase, the ATTD of P and Ca in the sorghum diet were increased by more than 20% and more than 10%, along with decreased chymotrypsin activity in the jejunum and ileum and increased trypsin activity in the ileum.	[88,89]
	1000 FTU/kg of microbial phytase	13.7 ± 1.3	The addition of phytase can significantly improve the digestibility of Ca and P in sorghum, but the application effect of phytase on sorghum is better than on corn.	[90]
	0, 400, 800, 1200, and 1600 U/kg of aspergillus niger phytase/kg	21.6 and 24.8	Adding 400 U/kg of phytase can meet the needs of growing pigs, and the FCR is significantly higher than that of the group without adding enzyme preparation.	[91]
	250 mg/kg of coated compound proteases	23.4 ± 1.2	Protease increased the ATTD of CP by more than 8% and improved the feed efficiency of growing pigs.	[92]
	360 mL/t (1650 U/g of amylase and 30 U/mL of cellulase)	46.1	Finishing pigs fed diets with enzymes showed some positive trends for improved growth performance.	[93]
Particle size	900, 700, 500, and 300 µm	5.3	The ADG and FCR increased linearly as the particle size was decreased to 300 µm.	[79]
	1262 and 471 µm	30	Reducing the feed size can increase the ileal digestibility of the DM (12.2%), starch (16.0%), and GE (12.6%).	[80]
	724, 573, and 319 µm	46.8 ± 1.24	As the particle size of sorghum was reduced from 724 to 319 µm, the ADG was not affected but the FCR increased; the dressing percentage tended to improve.	[2]
Conditioning temperature	65, 70, 75, 80, and 85 °C	21.40 ± 0.39	Temperatures of 75 and 80 °C were suitable conditioning temperatures for sorghum-based diets for pigs; the number of beneficial bacteria in the intestine and feces was increased; and the number of pathogenic bacteria was inhibited.	[77]
Extrusion	—	14.2 ± 0.9	The extruded process increased the DE (6.0%), ME (4.9%), starch AID (5.8%), and CP SID (5.5%).	[85]
Steam-flaking	—	primiparous sows	The digestibility of nitrogen and the GE of sows fed expanded sorghum increased, and the excretion of the DM and nitrogen in feces decreased.	[94]
Extrusion	—	5.9	The extrusion of sorghum improved the growth performance of pigs, and the feed conversion rate increased by 9.7%.	[95]
Expansion	—	58	The pellet durability index of extruded particles was increased by 10%, and the FCR had no significant change.	[96]

**Table 5:** Effects of sorghum on growth performance of pigs.

Body Weight (kg)	Corn/Sorghum (%)	ADFI (g/d)	ADG (g/d)	FCR	References
7.2	30:30	457	295	1.54	[21]
	0:60	734	451	1.61	
7.3	29:29	469	298	1.56	[4]
	0:57	784	473	1.64	
8.8	40:20	684	415	1.64	[97]
	20:40	676	408	1.65	
	0:60	663	401	1.66	
9.7	0:59	851	557	1.52	[88]
9.4	0:66	1210	648	1.86	[98]
	44.22:21.78	1230	613	2.00	
	22.4:43	1220	610	1.96	
	66:0	1250	620	2.02	
18.4	0:65—72	1443	770	1.87	[66]
54.4	0:72—83	2670	950	2.78	[86]

## Data Availability

No data were used for the research described in this article.
